# Childhood traumas as a risk factor for HIV-risk behaviours amongst young women and men living in urban informal settlements in South Africa: A cross-sectional study

**DOI:** 10.1371/journal.pone.0195369

**Published:** 2018-04-06

**Authors:** Andrew Gibbs, Kristin Dunkle, Laura Washington, Samantha Willan, Nwabisa Shai, Rachel Jewkes

**Affiliations:** 1 Gender and Health Research Unit, South African Medical Research Council, Pretoria, South Africa; 2 Health Economics and HIV/AIDS Research Division (HEARD), University of KwaZulu-Natal, Durban, South Africa; 3 Project Empower, Durban, South Africa; 4 School of Public Health, University of Witwatersrand, Johannesburg, South Africa; Stellenbosch University, SOUTH AFRICA

## Abstract

Childhood traumas, in the form of physical, sexual, and emotional abuse and neglect, are globally widespread and highly prevalent, and associated with a range of subsequent poor health outcomes. This study sought to understand the relationship between physical, sexual and emotional childhood abuse and subsequent HIV-risk behaviours amongst young people (18–30) living in urban informal settlements in Durban, South Africa. Data came from self-completed questionnaires amongst 680 women and 677 men comprising the baseline of the Stepping Stones and Creating Futures intervention trial. Men and women were analysed separately. Logistic regression models assessed the relationship between six HIV-risk behaviours and four measures of trauma: the form of trauma, the severity of each trauma, the range of traumas, and overall severity of childhood trauma. Childhood traumas were incredibly prevalent in this population. All childhood traumas were associated with a range of HIV-risk behaviours. This was for the ever/never trauma, as well as the severity of each type of trauma, the range of trauma, and overall severity of childhood trauma. Despite the wider harsh contexts of urban informal settlements, childhood traumas still play a significant role in shaping subsequent HIV-risk behaviours amongst young people. Interventions to reduce childhood traumas for populations in informal settlements need to be developed. In addition, trauma focused therapies need to be considered as part of wider HIV-prevention interventions for young adults.

**Trial registration:** ClinicalTrials.gov NCT03022370

## Introduction

Childhood traumas, in the form of physical, sexual, and emotional abuse and neglect, are globally widespread and highly prevalent [1, 2]. A systematic review of population-based studies estimated that amongst 2–17 year olds, the minimum prevalence of past-year physical abuse was at least 50% for Africa, Asia, and Northern America and over 30% for Latin America [1]. For youth aged 2 to 14, estimated prevalence of past-year violence was up to 80% in Africa [1]. In South Africa, a multi-community sample of children aged 10–17, found prevalence of lifetime physical abuse was 56.3%, emotional abuse 35.5% and sexual abuse 9% [3]. Overall, in the South African multi-community sample of children just over two-thirds (68.9%) reported any lifetime abuse and a quarter (27.1%) reported experiencing multiple forms of abuse [3]. In a young adult rural population (15–26) in South Africa, 39.1% of women and 16.7% of men had experienced sexual abuse, while 89.3% of women and 94.4% of men had experienced physical punishment as children [4].

As well as being a human rights violation, experiences of childhood traumas have short and long-term impacts on people’s health. Women and men who have experienced childhood traumas are more likely to report a range of sexual risk behaviours [5], including more likely to trade sex for cash [6], experience or perpetrate intimate partner violence (IPV) [7, 8], engage in risky alcohol use, and drug use [4, 5], and have more sex partners [6]. In South Africa, a longitudinal study showed those who experienced childhood traumas were also more likely to acquire HIV [4].

Globally, urban informal settlements are rapidly growing as the global population urbanises [9]. Urban informal settlements, while providing substantial opportunities associated with urban economies, are also settings with significant health challenges. Studies suggest that HIV prevalence and violence against women in informal settlements are higher than in formal settlements [10–12]. There is some suggestion that urban residency may also increase experiences of childhood traumas [13]. Despite this, there is little evidence about the relationship between childhood traumas and current HIV-risk behaviours amongst residents of informal settlements.

In this paper, we seek to describe the associations between sexual, physical, and emotional childhood traumas amongst women and men resident in informal settlements in eThekwini, South Africa and their subsequent HIV-risk behaviours. We draw on data from the baseline of the Stepping Stones and Creating Futures trial, a cluster randomized control trial (RCT) assessing the impact of this gender transformative and livelihood strengthening intervention on women’s experience, and men’s perpetration, of IPV [14]. We assess whether experience of each type of childhood trauma is independently associated with HIV-risk; whether there is a dose response for each type of childhood trauma and HIV-risk; whether the range of childhood traumas experienced is independently associated with HIV-risk; and whether the overall severity of childhood trauma is independently associated with HIV-risk.

## Methods

Data are drawn from N = 680 women and N = 677 men who participated in the baseline assessment for the Stepping Stones and Creating Futures RCT, carried out in urban informal settlements near Durban, South Africa between September 2015 and September 2016.

Women and men between 18 and 30, who were out of school, not in formal work and resident in an informal settlement, were eligible to participate. We worked with Project Empower, a local implementing agency, experienced in informal settlement interventions, to identify potential participants through community meetings and snow-ball sampling. The study comprised 34 clusters with 19–21 women and 16–22 men per cluster [14]. At recruitment, participants were not blinded to study arm. Those in the intervention arm received R100 (~US$7) and those in the control arm received R300 (~US$21) for completion of the baseline survey instrument. Ethical approval for the study was received from the University of KwaZulu-Natal and the South African Medical Research Council. Further information on procedures is described elsewhere [14].

### Data collection

Once enrolled into the study, participants self-completed structured questionnaires. Questionnaires were on study provided cellphones, and available in isiZulu, Xhosa, and English and had built in logic and skip patterns. Questionnaires had been tested prior to roll-out with young people from informal settlements, and young people were confident in using cellphones and this approach to data collection. If literacy was an issue, same sex trained fieldworkers were able to support questionnaire completion, through face-to-face interviews with participants entering the data, and interviewers reading out questions. Few participants (<5%) required this support, and as such, it was unlikely to influence responses given the small number of cases.

### Measures

Six variables, which have been associated with HIV-risk in a range of studies from across Africa, were identified as outcome variables for the analysis.

Transactional sex in the past year with a casual or once-off sexual partner. Five questions asked about whether a woman had sex with a man because she expected to receive, or did receive a range of items, including cash, a place to stay, drugs, school fees, airtime, or anything else she could not afford [15]. One, or more, positive responses to any of the five questions was coded as engaging in transactional sex. Men were asked whether they thought a woman had sex with them because she expected to receive items [16].

Sexual partners in past year. We asked three questions about sexual partners in the past year. We asked about the number of main sexual partners, casual sexual partners or *khwapheni*, and once-off sexual partners. In each instance, a cutoff score of 4 or more was used in the logistic regression and analysis kept each type of sex partner separate.

Condom use at last sex. A single question asked about condom use in last sex act. Responses were either yes or no.

Physical and/or sexual IPV perpetration (men) and experience (women) in the past year. Eight items, five physical IPV and three sexual IPV, were asked. Men were asked about perpetration and women about experience of IPV. Questions were based on the WHO multi-country survey on violence against women [17] and adapted and extensively tested in South Africa [18]. Responses for each item were never, once, few, and many. A positive response to any of the eight items led to a person being coded as having perpetrated IPV (men) and experienced IPV (women) in the past year.

Non-partner sexual violence perpetration (men) and experience (women) in the past year. Six items asked about women’s experience and men’s perpetration of non-partner sexual violence, including gang rape. Scales had been developed and extensively tested in South Africa [19]. Responses were never, once, few and many.

Harmful alcohol use. Alcohol use was assessed using the Alcohol Use Disorder Identification Test (AUDIT) scale. Ten items asked about past year alcohol use, with scores summed. A cut-off score of 8 or more indicated potential harmful alcohol use [20], and the same cut-off score was used for women and men.

Childhood traumas were the exposure variable. Eleven items asked about childhood traumas and were based on the on the short form of the Childhood Trauma Questionnaire [21] and modifications already undertaken in South Africa [4]. As all participants were 18 or older, participants were asked, “before I reached 18 I…” Five items asked about emotional abuse and neglect. Three items asked about physical abuse and witnessing of violence. Three items asked about experiencing sexual abuse. Responses were on a four-point Likert scale, never, sometimes, often, very often.

Childhood traumas were summarized in four ways. First, each type of trauma (emotional, physical, sexual) was dichotomized into a never/ever binary. Second, a straight score of severity for each type of trauma was created through simple summation of scores for individual items. Third, the range of traumas was assessed by classifying participants into those who had experienced no trauma, only one type, two types, or all three types. Finally, a direct summation of all items provided an overall childhood trauma severity score.

Socio-demographic measures includes age, educational level including whether a person had passed matric (high school leaving), and household food insecurity. Depression was assessed using The Centre for Epidemiologic Studies Depression Scale (CES-D) scale [22] and had been used in South Africa previously [23, 24]. Twenty items asked about depressive symptoms in the past week, with responses ranging from never to everyday (α = 0.88).

### Analysis

Men and women were analysed separately, and analyses included adjustment for cluster sampling. Descriptive statistics for the socio-demographic measures and each HIV-risk behaviour were first constructed. We then compiled descriptive statistics of the different forms of childhood traumas, including summaries for each type and the range of traumas.

For analysis, Gaussian regression models, reporting odds ratios and adjusting for clustering, age education, current food security, earnings, depressive symptoms, length of time living in community and study arm, were constructed for each HIV-risk behaviour. Model 1 examined whether a person had experienced childhood trauma (emotional, physical, sexual) at all and whether it was significantly associated with each HIV-risk variable. In model 2, each childhood trauma was treated as a score to indicate the severity of the trauma and assessment was made as to whether this independently predicted each HIV-risk behaviour. Model 3 assessed whether the range of traumas experienced was independently associated with each HIV-risk behaviour. Model 4 looked at overall severity of childhood trauma and its relationship to each HIV-risk behaviour.

## Results

In total 680 women and 677 men were recruited into the study. There were few differences between women and men in terms of age and education level (Table 1). More men than women reported HIV-risk behaviours, particularly transactional sex with a causal or once-off partner, and the number of all sexual partners in the past year. Condom use in the last year was similar, as was IPV experience/ perpetration and non-partner sexual violence experience/perpetration. Men reported higher levels of harmful alcohol use than women (43.3% and 23.1% respectively). Mean AUDIT scores for men were 7.8 (min-max scores 0–40), and for women 4.3 (min-max scores 0–38).

**Table 1:** Distribution of socio-demographic characteristics and HIV-risk behaviours amongst women and men.

	Women (n = 680)	Men (n = 677)
Socio-demographics	**n(%)**	**n(%)**
Age: 18–19	84(12.4)	70(10.3)
20–24	325(47.8)	355(52.4)
25–30	271(39.9)	252(37.2)
Education Primary only	55(8.1)	77(11.4)
Secondary (no matric)	419(61.6)	393(58.1)
Matric or beyond	206(30.3)	207(30.6)
HIV-risk behaviours		
Transactional sex past 12m	275(42.6)	338(50.7)
4 or more main sexual partners past 12m	61(9.5)	156(23.4)
4 or more causal sexual partners past 12m	19(3.0)	122(18.3)
4 or more once off sexual partner past 12m	13(2.0)	129(19.3)
Condom use at last sex	320(54.1)	395(60.3)
IPV experience/perpetration past 12m	443(65.2)	384(56.9)
Non-partner sexual violence experience/perpetration past 12m	224(32.9)	262(38.8)
Harmful alcohol use	157(23.1)	294(43.4)
Mean alcohol use scores (range)	4.3(0–38)	7.8(0–40)

Overall prevalence of childhood traumas were high. Table 2 shows the distribution of different forms of childhood abuse. Emotional abuse was very common with almost three-quarters of women (72.4%) and just over three-quarters of men (77.9%) reporting emotional abuse or neglect growing up. All types of emotional abuse were common, with the exception of parents being “too drunk or drugged to look after me”, where only around one quarter of women and men reported this. Physical abuse and witnessing of violence in the home was common and reported by about three quarters of participants. The most common form of physical abuse was being beaten with a belt, stick, or something hard, with about two-thirds of women and men reporting having experienced this at least once. Sexual abuse was the least form of childhood trauma, although still very high. One third of women (36.0%) and almost a half of men (47.9%) reported at least one type of sexual abuse. Experiencing more than one form of childhood abuse was also common. For women, 39.1% reported two forms of childhood abuse, and 27.7% three types of childhood abuse, while for men 31.6% reported two forms, and 40.2% all three forms of childhood abuse.

**Table 2:** Frequency and range of childhood traumas amongst women and men.

	Women				Men			
**Emotional abuse/neglect**	**Never, n(%)**	**Sometimes, n(%)**	**Often, n(%)**	**Very often, n(%)**	**Never, n(%)**	**Sometimes, n(%)**	**Often, n(%)**	**Very often, n(%)**
I lived in different households at different times	406(59.7)	169(24.9)	63(9.3)	42(6.2)	355(52.6)	203(30.1)	67(9.9)	50(7.4)
I was told I was lazy or stupid or weak by someone in my family	384(56.5)	180(26.5)	72(10.6)	44(6.5)	360(53.3)	220(32.6)	49(7.3)	46(6.8)
I was insulted or humiliated by someone in my family in front of other people	398(58.5)	185(27.2)	51(7.5)	46(6.8)	382(56.6)	197(29.2)	51(7.6)	45(6.7)
one or both of my parents were too drunk or drugged to take care of me	552(81.2)	69(10.2)	28(4.1)	31(4.6)	497(73.6)	101(15.0)	45(6.7)	32(4.7)
I spent time outside the home and none of the adults at home knew where I was	487(71.6)	139(20.4)	38(5.6)	16(2.4)	383(56.7)	213(31.6)	47(7.0)	32(4.7)
**Physical abuse/witnessing**								
I saw or heard my mother being beaten by her husband or boyfriend	476(70.0)	128(18.8)	39(5.7)	37(5.4)	439(65.0)	143(21.2)	54(8.0)	39(5.8)
I was beaten at home with a belt or stick or whip or something else which was hard	262(38.5)	275(40.4)	72(10.6)	71(10.4)	223(33.0)	289(42.8)	88(13.0)	75(11.1)
I was beaten so hard at home that it left a mark or bruise	428(62.9)	154(22.7)	55(8.1)	43(6.3)	412(61.0)	177(26.2)	44(6.5)	42(6.2)
**Sexual abuse**								
someone touched my buttocks or genitals or made me touch them when I did not want to	545(80.2)	102(15.0)	21(3.1)	12(1.8)	474(70.2)	139(20.6)	38(5.6)	24(3.6)
I had sex with a wo(man) who was more than 5 years older than me	533(78.4)	77(11.3)	46(6.8)	24(3.5)	460(68.2)	153(22.7)	44(6.5)	18(2.7)
I had sex with someone because I was threatened or frightened or forced	567(83.4)	65(9.6)	31(4.6)	17(2.5)	554(82.1)	82(12.2)	26(3.9)	13(1.9)
	**Never, n(%)**	**Ever, (n%)**			**Never, n(%)**	**Ever, (n%)**		
Emotional abuse ever	188(27.7)	492(72.4)			154(22.8)	521(77.9)		
Physical abuse ever	197(29.0)	483(71.0)			167(24.7)	508(75.3)		
Sexual abuse ever	435(64.0)	245(36.0)			352(52.2)	323(47.9)		
**Range of Abuse**	**None n(%)**	**One Form, n(%)**	**Two Forms, n(%)**	**Three Forms, n(%)**	**None n(%)**	**One Form, n(%)**	**Two Forms, n(%)**	**Three Forms, n(%)**
	102(15.0)	124(18.2)	266(39.1)	188(27.7)	78(11.6)	113(16.7)	213(31.6)	271(40.2)

For women, transactional sex in the past year was associated with ever experiencing emotional abuse, severity of emotional abuse, ever experiencing sexual abuse, and severity of sexual abuse (Table 3). Having four or more main sexual partners in the past year, and having four or more casual sexual partners in the past year were both associated with any sexual abuse, and severity of sexual abuse for women. Women reporting more than four once-off sexual partners in the past year were more likely to report ever being sexually abused. Non-condom use was associated with ever experiencing emotional abuse, and ever experiencing physical abuse. Past year IPV experience was associated with any emotional abuse, ever physical abuse, severity of physical abuse, any sexual abuse and severity of sexual abuse in childhood. Experience of non-partner sexual violence and harmful alcohol use were both associated with any emotional abuse, severity of emotional abuse, any physical abuse and severity of physical abuse, and any sexual abuse and severity of sexual abuse.

**Table 3:** Adjusted odds ratios for women and men for each HIV-risk behaviour and form of childhood trauma.

	**Emotional abuse ever (model 1)**	**Emotional abuse score (model 2)**	**Physical abuse ever (model 1)**	**Physical abuse score (model 2)**	**Sexual abuse ever (model 1)**	**Sexual abuse score (model 2)**	**Range of abuse (none-three, model 3)**	**Childhood trauma score (model 4)**
**Women**	**aOR(95%CI)**	**aOR(95%CI)**	**aOR(95%CI)**	**aOR(95%CI)**	**aOR(95%CI)**	**aOR(95%CI)**	**aOR(95%CI)**	**aOR(95%CI)**
Transactional sex	1.85(1.25–2.75)**	1.14(1.06–1.22)***	1.17(0.80–1.69)	1.06(0.97–1.16)	1.52(1.07–2.16)*	1.23(1.09–1.39)**	1.30(1.09–1.55)**	1.07(1.03–1.11)***
Main partners past12m	1.16(0.60–2.24)	1.06(0.96–1.17)	1.09(0.58–2.05)	1.04(0.91–1.20)	2.44(1.38–4.29)**	1.19(1.03–1.38)*	1.35(0.99–1.84)	1.04(0.99–1.10)
Casual partners past12m	1.87(0.50–7.00)	1.16(0.98–1.38)	0.99(0.33–2.98)	1.12(0.90–1.40)	6.17(2.05–18.51)**	1.25(1.00–1.56)*	2.00(1.08–3.71)*	1.09(1.00–1.18)
Once off sexual partners past12m	1.85(0.38–9.08)	1.11(0.90–1.37)	1.10(0.28–4.33)	1.03(0.77–1.38)	4.74(1.34–16.78)*	1.13(0.83–1.53)	1.89(0.90–3.96)	1.05(0.94–1.17)
Condom use at last sex	0.68(0.46–1.01)*	0.97(0.91–1.04)	0.60(0.41–0.88)**	0.97(0.89–1.06)	1.07(0.75–1.52)	0.98(0.88–1.10)	0.84(0.71–1.00)	0.99(0.95–1.02)
IPV (experience)	1.76(1.21–2.56)**	1.06(0.99–1.14)	2.16(1.51–3.10)***	1.13(1.02–1.25)**	1.44(1.00–2.08)*	1.17(1.02–1.34)*	1.46(1.23–1.74)***	1.05(1.01–1.10)**
Non-partner sexual violence (experience)	2.67(1.69–4.21)***	1.22(1.14–1.31)***	1.66(1.11–2.48)**	1.15(1.05–1.26)**	2.71(1.90–3.88)***	1.33(1.18–1.50)***	1.81(1.48–2.21)***	1.11(1.07–1.15)***
Harmful alcohol use	2.74(1.61–4.68)***	1.19(1.11–1.29)***	1.96(1.21–3.19)**	1.14(1.04–1.26)**	2.26(1.51–3.39)***	1.30(1.15–1.46)***	1.80(1.42–2.28)***	1.10(1.06–1.14)***
	**Emotional abuse ever (model 1)**	**Emotional abuse score (model 2)**	**Physical abuse ever (model 1)**	**Physical abuse score (model 2)**	**Sexual abuse ever (model 1)**	**Sexual abuse score (model 2)**	**Range of abuse (none-three, model 3)**	**Childhood trauma score (model 4)**
**Men**	**aOR(95%CI)**	**aOR(95%CI)**	**aOR(95%CI)**	**aOR(95%CI)**	**aOR(95%CI)**	**aOR(95%CI)**	**aOR(95%CI)**	**aOR(95%CI)**
Transactional sex	1.72(1.15–2.58)**	1.18(1.10–1.26)***	1.36(0.93–1.98)	1.09(1.00–1.19)*	2.90(2.07–4.05)***	1.52(1.32–1.74)***	1.54(1.30–1.84)***	1.09(1.06–1.13)***
Main partners past12m	1.24(0.77–1.99)	1.11(1.03–1.19)**	1.19(0.76–1.85)	1.11(1.01–1.22)*	2.63(1.76–3.91)***	1.29(1.15–1.46)***	1.39(1.13–1.71)**	1.07(1.03–1.11)**
Casual partners past12m	1.79(1.01–3.17)*	1.14(1.05–1.23)**	1.59(0.95–2.69)	1.13(1.02–1.25)*	2.57(1.65–4.00)***	1.31(1.15–1.48)***	1.57(1.23–2.01)***	1.08(1.04–1.12)***
Once off sexual partners past12m	2.04(1.14–3.66)*	1.10(1.02–1.19)*	1.65(0.98–2.77)	1.10(0.99–1.21)	2.82(1.81–4.40)***	1.32(1.16–1.50)***	1.67(1.31–2.14)***	1.06(1.02–1.11)**
Condom use at last sex	0.85(0.56–1.28)	0.95(0.89–1.01)	0.58(0.39–0.86)**	0.95(0.87–1.03)	0.87(0.63–1.21)	0.99(0.89–1.10)	0.84(0.71–1.00)*	0.98(0.95–1.01)
IPV (perpetration)	2.02(1.36–2.99)***	1.14(1.06–1.22)***	1.91(1.32–2.77)**	1.16(1.06–1.27)**	2.32(1.67–3.22)***	1.39(1.21–1.58)***	1.60(1.35–1.89)***	1.09(1.05–1.13)***
Non-partner sexual violence (perpetration)	2.21(1.42–3.45)***	1.17(1.10–1.25)***	1.85(1.23–2.77)**	1.15(1.05–1.25)**	3.98(2.80–5.67)***	1.68(1.47–1.92)***	1.92(1.58–2.34)***	1.11(1.07–1.15)***
Harmful alcohol use	1.97(1.29–3.00)***	1.23(1.06–1.20)***	2.11(1.42–3.15)***	1.15(1.06–1.26)**	1.74(1.25–2.43)**	1.10(0.99–1.22)	1.50(1.26–1.79)***	1.06(1.03–1.10)***

All models control for: age, education, food security, earnings, depression, intervention arm, length of time living in community

Levels of significance:

*p<0.05;

**p<0.01;

***p<0.0001

For men, transactional sex in the past year was associated with ever experiencing emotional abuse and severity of emotional abuse, severity of physical abuse, and any sexual abuse, and severity of sexual abuse. Four or more main sexual partners in the past year was associated with severity of emotional abuse, any physical abuse, severity of physical abuse, any sexual abuse and severity of sexual abuse. Four or more casual sexual partners in the past year was associated with any emotional violence and severity of emotional violence, severity of physical violence, and any sexual abuse, and severity of sexual abuse. Four or more once-off sexual partners was associated with any emotional abuse, severity of emotional abuse, any physical abuse, and any sexual abuse, and severity of sexual abuse. Not using a condom at last sex was associated with any experience of physical abuse. Perpetration of IPV and non-partner sexual violence and harmful alcohol use were all associated with ever experiencing emotional abuse, severity of emotional abuse, ever physical abuse, severity of physical abuse, and ever sexual abuse. IPV perpetration, and non-partner sexual violence perpetration was also associated with severity of sexual abuse.

For women the range of childhood abuse experienced was significantly associated with transactional sex, four or more main sexual partners, four or more causal sexual partners, experiencing IPV, non-partner sexual violence, not using a condom use at last sex and harmful alcohol use. The overall severity of childhood trauma for women was associated with transactional sex, four or more casual partners in the past year, past year experience of IPV, non-partner sexual violence, and harmful alcohol use. For men, the range of childhood abuse experienced and overall childhood trauma scores had similar associations. They were both independently associated with transactional sex, four or more main partners, casual partners or once off partners, and IPV and non-partner violence perpetration, and harmful alcohol use. The range of childhood abuse experienced for men was also independently associated with not using a condom at last sex.

## Discussion

The experience of violence and abuse during childhood for this vulnerable, and relatively young population, was exceedingly high: 75.0% of women and 88.4% of men reported experiencing any childhood trauma. All forms of childhood trauma were associated with a range of current HIV-risk behaviours, and this was true for ever experiencing a variety of types of trauma, the severity of trauma, the range of traumas experienced, and overall severity of childhood trauma.

The high prevalence of childhood trauma observed here is somewhat higher than other studies in South Africa, suggesting that childhood abuse remains a major challenge that needs tackling in South Africa. It also reinforces the growing evidence suggesting informal settlements are particular sites of vulnerability for childhood trauma [13] as well as sites of poor health more widely [11], and yet under-resourced in terms of access to services and less focused on by interventions [10, 12, 25]. The models adjusted for length of residency in the community, but did not specifically assess where participants had grown up, nor where they had experienced the childhood traumas they reported.

For men, there was a much higher prevalence of sexual abuse in childhood than in many other studies in South Africa. In this study, 47.9% of men reported any form of childhood sexual abuse. This is much higher than a younger, rural cohort, where 16.7% reported any sexual abuse [4], and 9% in a much younger (10–17) probability sample [3]. While in a representative household sample of men in South Africa, 9.6% reported any sexual victimisation (rape) by a man [26]. However, the rate of sexual abuse seen is only slightly higher than a school-based survey where 36.8% of male students reported any form of sexual abuse [27]. The high levels of reported sexual abuse was not driven by any single item; 29.8% reported being touched against their will, 31.9% reported sex with woman 5 years older, and 17.9% reported being raped and coerced into sex, suggesting that it was not an issue with translation or comprehension of questions.

The analysis suggests that broadly for women and men, all forms of childhood traumas are associated with current HIV-risk behaviours. This relationship holds true whether the analysis is looking at ever experience of different forms of childhood trauma, or the severity of childhood traumas experienced. This reflects a growing body of global research that also outlines the close relationship between childhood trauma and HIV-vulnerability for women and men [4–6, 28].

There was some variation in the relationships across women and men however. For women and men sexual abuse was independently associated with all HIV-risk behaviours (apart from condom use), reflecting the fact that sexual abuse seems to be a major driver of long-term health impact. For women, the number of past year sex partners tended not to be as strongly associated with physical or emotional abuse, or the range or overall severity of childhood trauma, but this was not the case with men. This may reflect different approaches to internalizing and externalizing of behaviours, whereby there is a tendency for men to externalize traumatic behaviours, while women internalize following traumatic experiences [28].

It was also evident from the analysis that the range of childhood trauma (model 3) and the cumulative severity of childhood trauma (model 4) were both independently associated with HIV-risk. In other populations, including those experiencing significant trauma during adulthood, a similar relationship is seen [28–30], suggesting that the impact of childhood traumas continues to shape people’s lives over and above any subsequent traumatic experiences. Furthermore, approximately two-thirds of women and men experienced two or more types of trauma, highlighting the close overlap between different trauma types and that there is likely to be a cumulative impact.

The analysis also highlighted how women and men who experienced physical abuse or witnessed violence in their home were more likely to perpetrate IPV (men) or experience it (women) later in life. This co-occurrence of physical abuse and witnessing violence in childhood is increasingly recognized as a key driver of IPV [31] and has been seen in multiple settings globally [8, 31].

The data suggested that there were weaker relationships between childhood traumas and condom use at last sex for women and men. For women not using a condom at last sex was only associated with ever emotional abuse, ever physical abuse, and the range of abuse experienced. While for men, it was only associated with ever physical abuse and the range of abuse experienced. There are a number of possible explanations for the relatively weak associations compared to other HIV-risk behaviours examined. First, we did not control for type of partner that people reported last having sex with. Condom use amongst couples trying to get pregnant, in long-term relationships, or women on long-term contraceptives will decrease [32, 33]. Second, condom use is often poorly reported in surveys, and as such these relationships are poorly understood.

The exceedingly high levels of sexual abuse amongst this sample, alongside the particularly consistent and strong relationship between sexual abuse and HIV-risk behaviours in this population, as well in other studies, highlights sexual abuse in childhood as a key driver of poor health [6, 34] and suggests there is need for further research on this topic. At a very basic level, understanding why the levels of sexual abuse in the population are so high is critical. In addition, this study did not ask about the sex of perpetrators of sexual abuse (apart from in one item). There is research in South Africa showing that boys and young men experience sexual abuse by women as well as men [26, 35]. There may be important differences by sex of perpetrator in terms of prevalence, and long-term impacts. Understanding the prevalence, forms and impacts of childhood sexual abuse amongst young men and boys remains a key research challenge to enable a meaningful response to be constructed.

There are a number of limitations to the study. Data are cross-sectional and as such relationships may be bi-directional, and longitudinal studies are required to establish the temporality of associations. The retrospective nature of questions may mean reporting of childhood experiences is weaker, and there may be some level of confusion around when specific acts occurred i.e. before or after the age of 18 for some participants. Studies suggest that recall bias may lead to an under-reporting of events in childhood [36], as such, the impact of retrospective reporting of childhood traumas on the analysis is unclear, again suggesting the need for prospective studies. Finally, the sample were self-selecting into an intervention evaluation and are therefore not representative of the population of young people living in urban informal settlements. Inclusion criteria mean those in formal employment or school were not included in the study.

The analysis highlights the importance of reducing all forms of childhood traumas as a way to reduce subsequent HIV-risk amongst young women and men. An increasing number of interventions have shown the potential to reduce harsh parenting across the global south [37] and modifying and implementing these in informal settlements remains a critical challenge. Another response to this analysis is to consider how the range of current HIV and IPV-prevention interventions already being implemented can include components on reducing the traumatic effects of childhood trauma as part of their wider intervention. A range of trauma-focused cognitive behavioural interventions [38, 39] may have an important role to play in achieving this.

## Conclusion

Despite the huge challenges for people living in informal settlements such as poverty, gender inequalities, and lack of access to services, which independently shape HIV-risk behaviours, this study highlights that childhood traumas remained a salient driver of HIV-risk behaviours amongst young people. As the analysis showed, young people’s experiences of all forms of childhood trauma, specifically emotional abuse and neglect, physical abuse, and sexual abuse were exceedingly high in this population. Additionally, there were incredibly consistent findings across women and men, whereby childhood traumas, assessed in a wide variety of ways were consistently associated with a range of HIV-risk behaviours. As such, despite the immediacy of many of the problems of young people living in informal settlements, there remains a pressing need to reduce the experiences of childhood traumas and their long-term effects.

## Supporting information

S1 File Dataset for analysis of women. (CSV)

S2 File Dataset for analysis of men. (CSV)

## References

[1] Hillis S, Mercy J, Amobi A, Kress H. Global prevalence of past-year violence against children: a systematic review and minimum estimates. Pediatrics. 2016;137(3):1–13. 10.1542/peds.2015-4079 PMC6496958 26810785

[2] Unicef. Hidden in plain sight: A statistical analysis of violence against children. 2014.

[3] Meinck F, Cluver LD, Boyes ME, Loening-Voysey H. Physical, emotional and sexual adolescent abuse victimisation in South Africa: prevalence, incidence, perpetrators and locations. Journal of epidemiology and community health. 2016;70(9):910–6. 10.1136/jech-2015-205860 PMC5013157 26962202

[4] Jewkes RK, Dunkle K, Nduna M, Jama PN, Puren A. Associations between childhood adversity and depression, substance abuse and HIV and HSV2 incident infections in rural South African youth. Child abuse & neglect. 2010;34(11):833–41. 20943270 10.1016/j.chiabu.2010.05.002 PMC2981623

[5] Norman RE, Byambaa M, De R, Butchart A, Scott J, Vos T. The long-term health consequences of child physical abuse, emotional abuse, and neglect: a systematic review and meta-analysis. PLoS Med. 2012;9(11):e1001349 doi: 10.1371/journal.pmed.1001349 23209385 10.1371/journal.pmed.1001349 PMC3507962

[6] Arriola KR, Louden T, Doldren MA, Fortenberry RM. A meta-analysis of the relationship of child sexual abuse to HIV risk behavior among women. Child abuse & neglect. 2005;29(6):725–46. 15979712 10.1016/j.chiabu.2004.10.014

[7] Fonseka RW, Minnis AM, Gomez AM. Impact of adverse childhood experiences on intimate partner violence perpetration among Sri Lankan men. Plos One. 2015;10(8):e0136321 doi: 10.1371/journal.pone.0136321 26295577 10.1371/journal.pone.0136321 PMC4546656

[8] Fleming PJ, McCleary-Sills J, Morton M, Levtov R, Heilman B, Barker G. Risk Factors for Men’s Lifetime Perpetration of Physical Violence against Intimate Partners: Results from the International Men and Gender Equality Survey (IMAGES) in Eight Countries. Plos One. 2015;10(3):e0118639 doi: 10.1371/journal.pone.0118639 25734544 10.1371/journal.pone.0118639 PMC4348538

[9] UN Habitat. Habitat III Issue Paper 22—Informal Settlements. New York: UN Habitat, 2015.

[10] UNAIDS, Habitat U. Ending the AIDS Epidemic: The advantage of cities. Geneva: UNAIDS, 2015.

[11] WHO, UN-Habitat. Hidden Cities: Unmasking and overcoming health inequities in urban settings. Geneva: WHO and UN-Habitat, 2010.

[12] Gibbs A. Tackling gender inequalities and intimate partner violence in the response to HIV: moving towards effective interventions in Southern and Eastern Africa. African Journal of AIDS Research. 2016;15(2):141–8. doi: 10.2989/16085906.2016.1204331 27399043 10.2989/16085906.2016.1204331

[13] Meinck F, Cluver LD, Boyes ME, Mhlongo EL. Risk and protective factors for physical and sexual abuse of children and adolescents in Africa: A review and implications for practice. Trauma, Violence, & Abuse. 2015;16(1):81–107. 10.1177/1524838014523336 24648489

[14] Gibbs A, Washington L, Willan S, Ntini N, Khumalo T, Mbatha N, et al The Stepping Stones and Creating Futures intervention to prevent intimate partner violence and HIV-risk behaviours in Durban, South Africa: study protocol for a cluster randomized control trial, and baseline characteristics. BMC Public Health. 2017;17(1):336 doi: 10.1186/s12889-017-4223-x 28427380 10.1186/s12889-017-4223-x PMC5397780

[15] Dunkle K, Jewkes R, Brown HC, Gray GE, McIntryre JA, Harlow SD. Transactional sex among women in Soweto, South Africa: prevalence, risk factors and association with HIV infection. Soc Sci Med. 2004;59(8):1581–92. doi: 10.1016/j.socscimed.2004.02.003 15279917 10.1016/j.socscimed.2004.02.003

[16] Jewkes R, Morrell R, Sikweyiya Y, Dunkle K, Penn-Kekana L. Transactional relationships and sex with a woman in prostitution: prevalence and patterns in a representative sample of South African men. BMC public health. 2012;12(1):325. 22551102 10.1186/1471-2458-12-325 PMC3433345

[17] Garcia-Moreno C, Jansen HAFM, Ellsberg M, Heise L, Watts CH, Wo WM-CS. Prevalence of intimate partner violence: findings from the WHO multi-country study on women’s health and domestic violence. Lancet. 2006;368(9543):1260–9. doi: 10.1016/S0140-6736(06)69523-8 17027732 10.1016/S0140-6736(06)69523-8

[18] Jewkes R, Nduna M, Levin J, Jama N, Dunkle K, Khuzwayo N, et al A cluster randomized-controlled trial to determine the effectiveness of Stepping Stones in preventing HIV infections and promoting safer sexual behaviour amongst youth in the rural Eastern Cape, South Africa: trial design, methods and baseline findings. Trop Med Int Health. 2006;11(1):3–16. doi: 10.1111/j.1365-3156.2005.01530.x 16398750 10.1111/j.1365-3156.2005.01530.x

[19] Jewkes R, Nduna M, Jama Shai N, Dunkle K. Prospective study of rape perpetration by young South African men: incidence & risk factors. Plos One. 2012;7(5):e38210 doi: 10.1371/journal.pone.0038210 22675449 10.1371/journal.pone.0038210 PMC3365003

[20] Saunders JB, Aasland OG, Babor TF, De la Fuente JR, Grant M. Development of the alcohol use disorders identification test (AUDIT): WHO collaborative project on early detection of persons with harmful alcohol consumption-II. Addiction. 1993;88(6):791–804. 8329970 10.1111/j.1360-0443.1993.tb02093.x

[21] Bernstein DP, Stein JA, Newcomb MD, Walker E, Pogge D, Ahluvalia T, et al Development and validation of a brief screening version of the Childhood Trauma Questionnaire. Child abuse & neglect. 2003;27(2):169–90. 12615092 10.1016/s0145-2134(02)00541-0

[22] Radloff LS. The CES-D scale a self-report depression scale for research in the general population. Applied psychological measurement. 1977;1(3):385–401.

[23] Gibbs A, Govender K, Jewkes R. An exploratory analysis of factors associated with depression in a vulnerable group of young people living in informal settlements in South Africa. Global Public Health. Forthcoming:1–16. 10.1080/17441692.2016.1214281 27533487

[24] Nduna M, Jewkes RK, Dunkle KL, Shai NP, Colman I. Associations between depressive symptoms, sexual behaviour and relationship characteristics: a prospective cohort study of young women and men in the Eastern Cape, South Africa. Journal of the International AIDS Society. 2010;13(1):44. 21078150 10.1186/1758-2652-13-44 PMC2992477

[25] Gibbs A, Willan S, Misselhorn A, Mangoma J. Combined structural interventions for gender equality and livelihood security: a critical review of the evidence from southern and eastern Africa and the implications for young people. Journal of the International AIDS Society. 2012;15(Supp. 1):17362. 10.7448/IAS.15.3.17362 PMC3499786 22713350

[26] Dunkle KL, Jewkes RK, Murdock DW, Sikweyiya Y, Morrell R. Prevalence of consensual male–male sex and sexual violence, and associations with HIV in South Africa: A population-based cross-sectional study. PLoS medicine. 2013;10(6):e1001472 doi: 10.1371/journal.pmed.1001472 23853554 10.1371/journal.pmed.1001472 PMC3708702

[27] Artz L, Burton P, Ward C, Leoschut L, Phyfer J, Kassanjee R, et al. Optimus Study South Africa: Technical Report. Sexual Victimisation of Children in South Africa. Final Report of the Optimus Foundation Study. South Africa. Zurich* UBS Optimus Foundation; 2016.

[28] Closson K, Dietrich JJ, Nkala B, Musuku A, Cui Z, Chia J, et al Prevalence, type, and correlates of trauma exposure among adolescent men and women in Soweto, South Africa: implications for HIV prevention. BMC public health. 2016;16(1):1191 doi: 10.1186/s12889-016-3832-0 27884181 10.1186/s12889-016-3832-0 PMC5123224

[29] Anda RF, Felitti VJ, Bremner JD, Walker JD, Whitfield C, Perry BD, et al The enduring effects of abuse and related adverse experiences in childhood. European archives of psychiatry and clinical neuroscience. 2006;256(3):174–86. doi: 10.1007/s00406-005-0624-4 16311898 10.1007/s00406-005-0624-4 PMC3232061

[30] Suliman S, Mkabile SG, Fincham DS, Ahmed R, Stein DJ, Seedat S. Cumulative effect of multiple trauma on symptoms of posttraumatic stress disorder, anxiety, and depression in adolescents. Comprehensive psychiatry. 2009;50(2):121–7. doi: 10.1016/j.comppsych.2008.06.006 19216888 10.1016/j.comppsych.2008.06.006

[31] Fulu E, Miedema S, Roselli T, McCook S, Chan KL, Haardörfer R, et al Pathways between childhood trauma, intimate partner violence, and harsh parenting: findings from the UN Multi-country Study on Men and Violence in Asia and the Pacific. The Lancet Global Health. 2017;5(5):e512–e22. doi: 10.1016/S2214-109X(17)30103-1 28395846 10.1016/S2214-109X(17)30103-1

[32] Maharaj P, Cleland J. Risk perception and condom use among married or cohabiting couples in KwaZulu-Natal, South Africa. International family planning perspectives. 2005:24–9. 15888406 10.1363/3102405

[33] Cushman LF, Romero D, Kalmuss D, Davidson AR, Heartwell S, Rulin M. Condom use among women choosing long-term hormonal contraception. Family Planning Perspectives. 1998:240–3. 9782048

[34] Irish L, Kobayashi I, Delahanty DL. Long-term physical health consequences of childhood sexual abuse: A meta-analytic review. Journal of pediatric psychology. 2009: jsp118. 10.1093/jpepsy/jsp118 PMC2910944 20022919

[35] Sikweyiya Y, Jewkes RK. Force and temptation: contrasting South African men’s accounts of coercion into sex by men and women. Culture, Health & Sexuality. 2009;11(5):529–41. 10.1080/13691050902912783 PMC2947024 19499390

[36] Williams LM. Recall of childhood trauma: a prospective study of women’s memories of child sexual abuse. Journal of consulting and clinical psychology. 1994;62(6):1167 7860814 10.1037//0022-006x.62.6.1167

[37] Knerr W, Gardner F, Cluver L. Improving positive parenting skills and reducing harsh and abusive parenting in low-and middle-income countries: A systematic review. Prevention science. 2013;14(4):352–63. doi: 10.1007/s11121-012-0314-1 23315023 10.1007/s11121-012-0314-1

[38] Murray LK, Skavenski S, Kane JC, Mayeya J, Dorsey S, Cohen JA, et al Effectiveness of trauma-focused cognitive behavioral therapy among trauma-affected children in lusaka, zambia: A randomized clinical trial. JAMA pediatrics. 2015;169(8):761–9. doi: 10.1001/jamapediatrics.2015.0580 26111066 10.1001/jamapediatrics.2015.0580 PMC9067900

[39] de Arellano MAR, Lyman DR, Jobe-Shields L, George P, Dougherty RH, Daniels AS, et al Trauma-focused cognitive-behavioral therapy for children and adolescents: Assessing the evidence. Psychiatric Services. 2014;65(5):591–602. doi: 10.1176/appi.ps.201300255 24638076 10.1176/appi.ps.201300255 PMC4396183

